# Current Management of Hyperkalemia in Non-Dialysis CKD: Longitudinal Study of Patients Receiving Stable Nephrology Care

**DOI:** 10.3390/nu13030942

**Published:** 2021-03-15

**Authors:** Silvio Borrelli, Luca De Nicola, Roberto Minutolo, Giuseppe Conte, Paolo Chiodini, Adamasco Cupisti, Domenico Santoro, Vincenzo Calabrese, Domenico Giannese, Carlo Garofalo, Michele Provenzano, Vincenzo Bellizzi, Luca Apicella, Giorgina Barbara Piccoli, Massimo Torreggiani, Biagio Raffaele Di Iorio

**Affiliations:** 1 Nephrology Unit, University of Campania “Luigi Vanvitelli”, 80138 Naples, Italy; dottsilvioborrelli@gmail.com (S.B.); roberto.minutolo@unicampania.it (R.M.); giuseppe.conte@unicampania.it (G.C.); carlo.garofalo@unicampania.it (C.G.); 2 Medical Statistics Unit, University of Campania “Luigi Vanvitelli”, 80138 Naples, Italy; paolo.chiodini@unicampania.it; 3 Department of Clinical and Experimental Medicine, University of Pisa, 56121 Pisa, Italy; adamasco.cupisti@med.unipi.it (A.C.); domenico.giannese@phd.unipi.it (D.G.); 4 Unit of Nephrology and Dialysis, Department of Clinical and Experimental Medicine, University of Messina, 98168 Messina, Italy; dsantoro@unime.it (D.S.); v.calabrese@outlook.it (V.C.); 5 Nephrology Unit, “Magna Graecia”, Department of Health Sciences, “Magna Graecia”, University of Catanzaro, 88100 Catanzaro, Italy; michiprov@hotmail.it; 6 Nephrology Unit, University Hospital “San Giovanni di Dio e Ruggi d’Aragona”, 84135 Salerno, Italy; vincenzo@bellizzi.eu (V.B.); lucaapicella@gmail.com (L.A.); 7 Nephrology Unit, San Luigi Hospital-University of Torino, 10100 Torino, Italy; gbpiccoli@yahoo.it; 8 Nephrology Unit, Centre Hospitalier Le Mans, 72037 Le Mans, France; maxtorreggiani@hotmail.com; 9 Division of Nephrology, Moscati Hospital, 83100 Avellino, Italy; br.diiorio@gmail.com

**Keywords:** potassium, hyperkalemia, diet, RAASI, CKD

## Abstract

Background: No study has explored the limitations of current long-term management of hyperkalemia (HK) in outpatient CKD clinics. Methods: We evaluated the association between current therapeutic options and control of serum K (sK) during 12-month follow up in ND-CKD patients stratified in four groups by HK (sK ≥ 5.0 mEq/L) at baseline and month 12: Absent (no-no), Resolving (yes-no), New Onset (no-yes), Persistent (yes-yes). Results: We studied 562 patients (age 66.2 ± 14.5 y; 61% males; eGFR 39.8 ± 21.8 mL/min/1.73 m^2^, RAASI 76.2%). HK was “absent” in 50.7%, “resolving” in 15.6%, “new onset” in 16.6%, and “persistent” in 17.1%. Twenty-four hour urinary measurements testified adherence to nutritional recommendations in the four groups at either visit. We detected increased prescription from baseline to month 12 of bicarbonate supplements (from 5.0 to 14.1%, *p* < 0.0001), K-binders (from 2.0 to 7.7%, *p* < 0.0001), and non-K sparing diuretics (from 34.3 to 41.5%, *p* < 0.001); these changes were consistent across groups. Similar results were obtained when using higher sK level (≥5.5 mEq/L) to stratify patients. Mixed-effects regression analysis showed that higher sK over time was associated with eGFR < 60, diabetes, lower serum bicarbonate, lower use of non-K sparing diuretics, bicarbonate supplementation, and K-binder use. Treatment-by-time interaction showed that sK decreased in HK patients given bicarbonate (*p* = 0.003) and K-binders (*p* = 0.005). Conclusions: This observational study discloses that one-third of ND-CKD patients under nephrology care remain with or develop HK during a 12-month period despite low K intake and increased use of sK-lowering drugs.

## 1. Introduction

In non-dialysis chronic kidney disease (ND-CKD), the burden of hyperkalemia (HK) is significant in terms of prevalence and patient survival even when serum potassium (sK) levels are only moderately increased [1,2]. Indeed, the recent individual-level data meta-analysis of the CKD Prognosis Consortium including more than 1 million subjects, followed for a mean of 6.9 years, demonstrated that cardiorenal risk was lowest with sK levels of 4–4.5 mEq/L and increased from the 5.0 mEq/L threshold across all eGFR strata [2]. Similar data have been shown in 2164 patients at discharge after decompensated heart failure [3]. Furthermore, the cost of providing care for patients with even moderate HK is double the cost of care for those whose potassium levels are normal [4,5].

Treatment of HK is therefore a key issue in the management of ND-CKD patients, with dietary education and correction of acidosis being the major interventions that are integrated by K resin prescription as adjuvant therapy. Poor sK control is multifactorial: it is caused not only by impaired renal function, leading to insufficient urinary potassium excretion in relation to intake and metabolic acidosis, but also by the widespread use of renin-angiotensin-aldosterone system inhibitors (RAASI) [6,7,8,9]. On the other hand, development of HK frequently prompts physicians to downtitrate or even stop RAASI therapy despite it being the first-choice therapy in ND-CKD for protecting patients from cardiovascular (CV) events and end stage kidney disease (ESKD) [10,11,12,13].

The importance of gaining insight into HK management becomes critical in outpatient nephrology clinics, which are the reference for care for patients with progressive CKD and where RAASI are regularly prescribed. However, no information on the therapeutic approach to HK in this setting is available. Early studies have provided data only on the prevalence and outcome of HK [14,15,16]. Those studies were also limited by the use of only a single sK test, which does not allow proper identification of true HK [1,17]. Only one study evaluated in renal clinics HK over two visits with 12-month interval [18]; that study reported that new onset or persistence of HK, defined as sK ≥ 5.0 mEq/L, significantly increased the risk of ESKD by 30%, while risk was neutral in patients with persistently absent or resolving HK. The study, however, did not provide data on the therapeutic approach to HK.

To fill this important gap in our knowledge, we designed an observational study lasting 12 months. The aim was to evaluate in CKD patients under nephrology care the association between current therapeutic options (dietary and pharmacological) and HK, defined as sK ≥ 5.0 mEq/L according to the several previous studies showing the association of these levels with hard outcomes [1,2,3,18]. Our data were obtained from six Italian nephrology clinics that share a similar multifactorial approach to ND-CKD, and where clinical and therapeutic data had been collected prospectively since referral. In the six clinics, nephrologists ensure continuity of care in the same patient since referral visit. This historical prospective design, where the aim of data collection is not established a priori, provides results on HK management representative of daily nephrology practice. We analyzed the main sK-lowering interventions implemented to date, that is in the absence of novel K binders which are not yet available in Italy, thereby allowing us to provide novel information on the current unmet needs in HK management in CKD clinics.

## 2. Materials and Methods

This multicenter observational study included consecutive patients, enrolled between January 2019 and January 2020, with CKD stage 1–5 (eGFR < 60 mL/min/1.73 m^2^ and/or proteinuria > 0.15 g/24 h lasting at least three months). The patients were not on dialysis or transplanted and had been referred at least 3 months prior to the beginning of the study to one of the six participating nephrology outpatient clinics (Nephrology Units at: “Luigi Vanvitelli”, University of Naples; Solofra Hospital-Avellino; Pisa University; Messina University; Hospital “San Giovanni di Dio e Ruggi d’Aragona”, Salerno; San Luigi Hospital, University of Torino). These Units all employ a similar multifactorial approach to treat ND-CKD, including prescribing decreased intake of salt (≤6 g/day), K (≤3.5 g/day) and protein (≤1 g/kg/day), coherent with well-established recommendations [19]. All subjects gave their informed consent to use their clinical data, and data collection was approved by ethical committee of University Vanvitelli (#472-04). We excluded patients with poor life expectancy (active malignancy, severe cirrhosis or heart failure) and/or acute kidney injury (30% increase in eGFR) in the 3 months prior to the baseline visit. We also excluded patients with no sK measurement after baseline visit.

### 2.1. Data Collection

Clinical, laboratory, and therapeutic data were collected at each study visit. To assess nutritional intakes, in addition to standard lab parameters, we measured 24-h urinary excretion of sodium (UNaV), potassium (UKV) and urea nitrogen (UUN): we estimated salt intake (g/day) by dividing UNaV by 17 [20], and protein intake by Mitch and Maroni formula [21]: (UUN (g/day) + weight (in kg) × 0.031) × 6.25. Urine collection was repeated if the 24-h creatinine excretion measured was outside the 60–140% range of the value calculated according to Dwyer and Kenler [22]. GFR was estimated using the Chronic Kidney Disease Epidemiology Collaboration (CKD-EPI) creatinine equation.

### 2.2. Statistical Methods

Continuous variables were reported as either mean ± standard deviation (SD) or median and interquartile ranges (IQR), according to their distribution. We grouped patients according to the presence of HK (sK ≥ 5.0 mEq/L) at baseline and month-12 visit: no hyperkalemia at either visit (absent); only at the first visit (resolving); only at the second visit (new onset); at both visits (persistent). Intra-group differences between baseline and final visit were assessed using the paired t-test (continuous variables) and Mc-Nemar Test (categorical variables). Inter-group differences were assessed with one-way analysis of variance (ANOVA) and the Kruskal Wallis test for parametric and non-parametric continuous variables, respectively; a chi squared test was used for categorical variables. We repeated analyses using higher sK level to define hyperkalemia (sK ≥ 5.5 mEq/L).

In a subgroup of 464 patients with available sK level also at the intermediate visit (6 ± 2 months), we estimated determinants associated with time-dependent sK by using a mixed effects regression model that accurately accounts for correlation between repeated measures [23]. Baseline confounders were included a priori in the analysis: age, sex, BMI, diabetes, prior CV disease, CKD stage, serum bicarbonate, UKV, protein and salt intake, RAASI, diuretics, bicarbonate supplements, and K-binders. We also tested the interaction of sK lowering treatment x time to assess whether the effect of treatment on sK level depends on time (treatment-by-time interaction). We tested multicollinearity by variance inflation factor (VIF; cut-off for multicollinearity VIF < 5) [24]. Data were analyzed by using STATA version 14.2 (4905 Lakeway Drive College Station, Texas 77845-4512 USA) and software R package.

## 3. Results

### 3.1. Clinical Features

Of the 624 eligible patients, selected on the basis of our inclusion criteria, we studied 562; 62 were in fact excluded for one of the following reasons: missing at month-12 visit (*n* = 27), start of chronic dialysis (*n* = 15); poor life expectancy (*n* = 15); recent AKI (*n* = 5). Of note, in included and excluded patients, we detected similar sK levels (4.7 ± 0.5 and 4.7 ± 0.6 mEq/L, respectively, *p* = 0.342), as well as prevalence of HK (32.7% vs. 27.4%, *p* = 0.395).

As reported in Table 1, the entire cohort was characterized by advanced age and high prevalence of diabetes and CV disease, as well as by advanced CKD (66.7% of the patients had CKD stage 3B-5) with hypertensive and diabetic nephropathies accounting for 57% of primary renal diseases. Overall, HK was detected at baseline in 184 patients (32.7%), of which the large majority (66.9%) had sK in the 5.0–5.49 mEq/L range while 24.4% patients had sK 5.5–5.99 and sK > 6.0 mEq/L was detected in only 8.7%. The prevalence of HK increased in parallel with CKD severity (sK ≥ 5.0 mEq/L was 5.3% in stage 1–2, 21.6% in stage 3A, 34.1% in stage 3B, 45.6% in stage 4, 54.0% in stage 5); similarly, the prevalence of sK ≥ 5.5 mEq/L increased by stage (1.3% in stage 1–2, 4.5% in stage 3A and 7.8% in 3B, 17.7% in stage 4 and 26.0% in stage 5).

The prevalence of HK in the entire cohort remained stable over 12 months of observation: (32.7% at baseline vs. 33.7% at month-12). After stratification by HK status, the “absent” subgroup accounted for 50.7% of the cohort and “resolving” was detected in 15.6%, whereas HK was “new onset” and “persistent” in 16.6% and 17.1%, respectively, for a total of 33.7% with sK ≥ 5.0 mEq/L at month 12. The main clinical features by HK status are described in Table 1. Patients with new onset or persistent HK had more advanced stages of CKD compared to those in the absent and resolving groups, while the distribution of primary kidney disease did not differ.

We also re-evaluated HK status using a higher threshold (sK ≥ 5.5 mEq/L). As expected, HK prevalence by this definition was lower at either basal and month-12 visit (10.3% and 8.2%, respectively; *p* = 0.122). Accordingly, we also observed a decreased prevalence of “persistent” (6.1%), “new onset” (2.1%) and “resolving” (8.7), whereas the prevalence of “absent” increased (83.1%). The main features of subgroups defined by this higher sK thresholds were overall similar to those of main analysis (Table S1).

The main changes of lab parameters at the two study visits are reported in Table 2. The entire population was characterized by a mean eGFR decline of −1.84 (95% CI: from −0.94 to −2.74 mL/min/1.73 m^2^), similar in the four subgroups (*p* = 0.191). The new-onset and persistent groups were characterized by lower levels of serum bicarbonate at baseline, associated with higher prevalence of metabolic acidosis; in these patients, bicarbonate levels improved to a similar degree at month-12 visit (+1.9 mEq/L, 95% CI 0.7–3.0 and +2.3 mEq/L, 95% CI 1.2–3.3), with a remarkable reduction in the frequency of acidosis.

### 3.2. Therapeutic Features

With regards to nutrient intakes, estimated using 24-h urine measurements (Table 2), salt intake was 8.8 ± 3.6 g/day and protein intake 1.0 ± 0.4 g/kg/day in the whole population at baseline. In the whole cohort, 24 h UKV (mEq/L) was 51.8 ± 23.9 at baseline and 53.4 ± 27.9 at month-12 visit (*p* = 0.370). We did not detect any significant difference for UUN, UNaV, and UKV between the two visits across groups at each visit. Time-average values of UUN and UKV were closely correlated (*r* = 0.244, *p* = 0.004).

**Figure 1:**
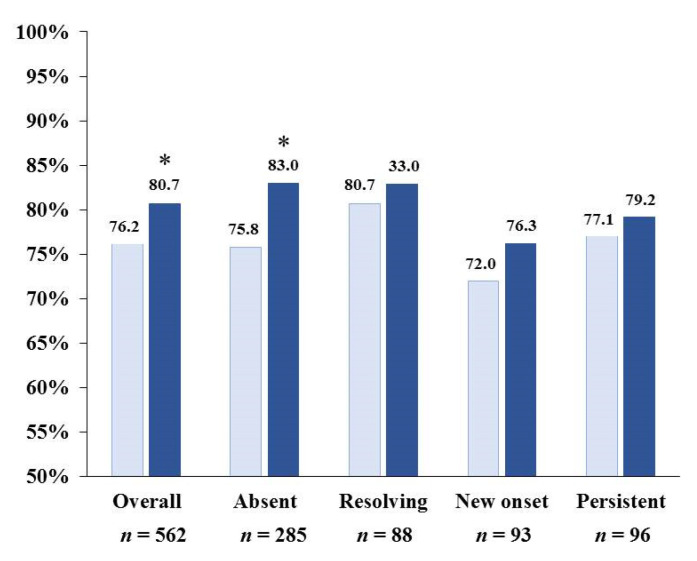
Use of Renin-Angiotensin-Aldosterone Inhibitors at baseline (light blue) and at 12-month visit (dark blue) in the whole cohort and in each HK subgroup (see text for definition of subgroups). * *p* < 0.05 vs. baseline.

At baseline, RAASI were found to be widely used; most patients received ACE inhibitors or Angiotensin II receptor antagonists while aldosterone receptor antagonists were prescribed in a minority of patients (*n* = 23; 4.1%). RAASI prescription increased in the whole cohort from 76.2% to 80.7% (*p* = 0.005). As shown in Figure 1, RAASI use was similarly high in the four groups at baseline (*p* = 0.588) and end of follow-up (*p* = 0.708). RAASI use increased numerically in all the subgroups although a significant change was detected only in the “absent” group (*p* = 0.007). Similar data were observed when considering the higher threshold value of sK to define HK (Figure S1).

**Figure 2:**
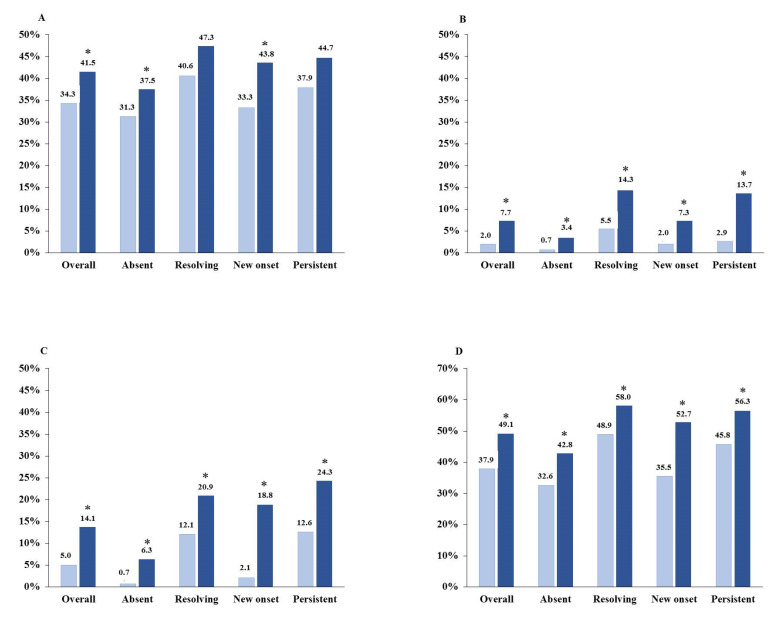
Anti-hyperkalemic therapy at baseline (light blue) and month-12 visit (dark blue): non-K sparing diuretics (panel **A**), K-binders (panel **B**), bicarbonate supplements (panel **C**), and at least one of the three drugs (panel **D**) in the whole cohort and in each HK subgroup (see text for definition and size of subgroups). * *p* < 0.05 vs. baseline.

In Figure 2, we report the prescription of different anti-hyperkalemia agents. In the whole cohort, the prescription of these agents significantly increased from baseline to month-12 visit (from 34 to 42% for non-K sparing diuretics, from 2% to 8% for K-binders and from 5% to 14% for bicarbonate supplements). Use of diuretic agents did not differ in the four categories at baseline (*p* = 0.296) and month-12 (*p* = 0.298), with a tendency to increase during follow-up in all groups that reached statistical significance only in the absent and new-onset groups (Panel A). Prescription of traditional K-binders and bicarbonate supplements increased in all groups (Panels B and C, respectively). Most patients with HK at baseline and/or month 12 were treated with at least one intervention by month-12 visit (Panel D), and the concomitant implementation of two or three different interventions significantly increased in “resolving” (from 8 to 21%), “new onset” (from 1 to 15%) and “persistent “(from 8 to 24%). Nonetheless, as many as 43.7% of “persistent” patients did not receive any drug at month-12 visit (Panel D).

Similar to the main analysis, when the sK ≥ 5.5 mEq/L threshold was used, we found no difference in protein and salt intake while the prescription of diuretics, K-binders, and bicarbonate supplements increased in all subgroups from basal to month-12 visit (Table S2).

### 3.3. Time-Dependent Serum K Levels and Interaction in the Whole Cohort

Mixed-effects regression analysis included 464 patients with available measurements also at month 6 (Table 3). Overall, sK levels remained unchanged over the 12 months of follow-up. Results demonstrated that higher sK levels over time were significantly associated with lower eGFR and diabetes and lower levels of serum bicarbonate, while diuretic use at baseline was associated with lower sK levels over time. Higher sK levels were also found to be associated with use of bicarbonate supplements and K-binders, whereas no association was found with RAASI.

**Figure 3:**
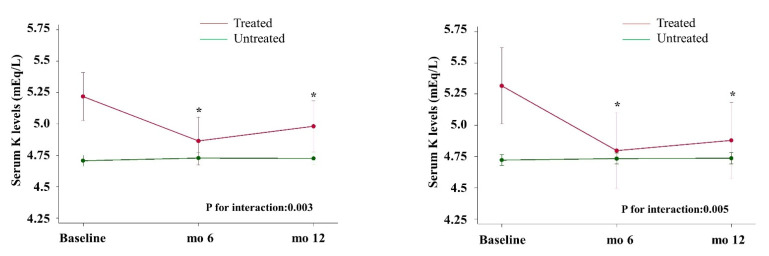
Treatment-by-time interaction between serum potassium levels in patients treated and not treated with bicarbonate supplements (left) and potassium-binders (right). * *p* < 0.05 vs. baseline.

When testing all potential interactions in this model, we observed a significant treatment-by-time interaction for bicarbonate supplements (*p* = 0.003) and K-binders (*p* = 0.005), as shown in Figure 3. Indeed, we detected a significant sK reduction in patients treated with K-binders at month 6 (−0.52; 95% CI −0.86, −0.18; *p* vs. baseline = 0.003), that persisted at month 12 (−0.44; 95% CI −0.78, −0.10; *p* vs. baseline = 0.012), whereas it remained unmodified in untreated patients (*p* = 0.638 and *p* = 0.587, respectively). Similarly, sK levels decreased in patients treated with bicarbonate supplements at month 6 (−0.36; 95% CI −0.57, −0.14, *p* vs. baseline = 0.001), and was found to be similar at month 12 (−0.24; 95% CI: −0.46, −0.01; *p* vs. baseline = 0.039), while no difference was detected in untreated patients (*p* = 0.425 and *p* = 0.509, respectively). No multicollinearity was detected.

## 4. Discussion

This multicenter observational study provides new insights into the effect of current nephrology management on HK in ND-CKD patients. Serum K ≥ 5.0 mEq/L was common in participating outpatient renal CKD clinics, detectable in 33.7% of patients after 12 months of nephrology follow up including nutritional and pharmacological treatment of hyperkalemia. These findings have important practical relevance as they provide for the first time a comprehensive picture of HK management in patients regularly followed in outpatient clinics primarily dedicated to ND-CKD management.

The results of nephrology care were evinced in slower CKD progression (mean eGFR loss < 2 mL/min in the year of observation) in patients where progressive CKD was expected due to the severity of disease at presentation (67% with CKD stage 3B-5 and a high prevalence of significant proteinuria, diabetes, and CV disease). This discrepancy is only apparent being, at least in part, related to the multifactorial treatment patients were given. Indeed, 24 h urine measurements suggested that intakes of salt and potassium were significantly lower in our ND-CKD patients than those reported in the general population as well as in hypertensive patients with preserved renal function from the same geographical area [25,26]. Similarly, protein intake was reduced to levels coherent with current recommendations in the guidelines [27]. Furthermore, more than 75% patients received RAASI therapy (Figure 1); this is at variance with the observed lower-than-expected use of these agents in an unselected CKD population where only 40% of the patients were treated [28]. On the other hand, this result agrees with the concept that RAASI should be preferred to other antihypertensive drugs and should not be discontinued even in advanced CKD, to preserve the cardiorenal benefits of therapy [10,11,12,13,29]. In this regard, a trend to a further increase in RAASI use was observed in all subgroups. Noteworthy, prescription of RAASI remained high even when patients were grouped by using the higher sK threshold (≥5.5 mEq/L) to define hyperkalemia (Figure S1). These findings confirm how important it is for nephrologists to tolerate moderate degrees of HK in order to maintain RAASI therapy. This attitude is supported by our recent study in a similar ND-CKD population showing that risk of ESKD significantly increases by 57% when moderate hyperkalemia associates with non-use of RAASI [18]. Nevertheless, in a recent large survey including 49,571 elderly patients from Canada (58% with eGFR < 60 mL/min/1.73 m^2^), all with sK ≥ 5.3 mEq/L under RAASI, discontinuation of RAASI was identified as the most frequent antihyperkalemic intervention (74%) [30]. The negative effect of stopping RAASI in HK patients has been recently highlighted by a registry-based study in 9222 patients with heart failure showing that association between HK and mortality risk disappeared after adjustment for RAASI withdrawal: discontinuation of RAASI did associate with 6 to 12-fold greater risk of death while high sK was no longer associated with outcome [31].

Longitudinal analyses provided further insights into sK control. Mixed-model regression showed that sK levels were higher over time in the presence of even moderate CKD, diabetes, and lower bicarbonate levels (Table 3). While the association between HK and low eGFR and/or metabolic acidosis is clear-cut, the relationship with diabetes is more complex, with several factors playing a role; it depends, in fact, not only on insulin deficiency and an increased use of RAASI, but also on hyporeninemic hypoaldosteronism ascribed to impairment to the juxtaglomerular apparatus, autonomic dysfunction and/or renal salt retention with dependent volume expansion suppressing renin synthesis [32].

In terms of therapeutic approach (Figure 2), prescription of traditional resins as adjuvant therapy, namely sodium polystyrene sulfonate and calcium polystyrene sulfonate, increased four-fold over 1 year of observation. Similarly, we registered an increased prescription of bicarbonate supplements, which made it possible to alleviate metabolic acidosis, as testified by the significant increment of serum bicarbonate levels (Table 2).

Significantly, in mixed-effects regression analysis, the use of specific K-lowering therapy was associated with higher sK levels; this finding is only apparently surprising because it is entirely due to indication bias, that is, the confounding effect dependent on the higher use of K-lowering drugs in patients with high sK. On the other hand, prescribing diuretics that promote kaliuresis was associated with lower sK levels; in this case, indication bias is not evident because diuretic use is based mainly on indications other than HK, that is, the volume-dependent hypertension of CKD.

Interestingly, the regression analysis also disclosed a treatment-by-time interaction between time-dependent sK levels and use of either K-binders or bicarbonate supplements. As illustrated in Figure 3, sK decreased in HK patients treated with K-binders, as well as in those receiving bicarbonate supplements, suggesting the efficacy of these interventions when tolerated by patients. About 44% of patients with persistent HK, however, remained untreated; it is likely that the low tolerability of current therapies played a major role. In fact, bicarbonate supplementation has gastrointestinal effects and sodium load remains a concern, and new agents are being developed to treat metabolic acidosis in CKD patients without administering extra amounts of sodium [33].

Similarly, the use of traditional potassium binders remains low, given the concerns about side effects, especially the risk of gastrointestinal events, some serious enough to require hospitalization [34,35]. As further support to this assumption, the recent large Canadian survey has evidenced that among hyperkalemic patients, those prescribed traditional K-binders were only 1% of cohort and this prescription, at variance with RAASI withdrawal, did not prevent subsequent episodes of high sK [30].

Of note, we can reasonably hypothesize that the phenomenon of “therapeutic inertia” was limited in our HK patients because we observed a significant intensification of therapy from baseline to end of observation, with increased prescription of simultaneous multiple anti-hyperkalemic interventions.

We measured 24 h urinary K excretion to have a rough estimate of dietary potassium intake since the two parameters have been found to be correlated in general population [36,37]. In the presence of normal renal function, a high potassium intake (>3.5 g/day) is recommended to lower CV risk [38], whereas an intake not greater than 3 g per day is recommended in patients with reduced kidney function [19,27,39]. In our cohort, urinary K may indicate an intake of about 2 g/day of potassium, that is well below the recommended level [19,27,39]. The lack of association disclosed by the mixed effect analysis further confirms that K intake does not play a major role in the control of chronic HK in ND-CKD patients because of the predominant role of low GFR, acidosis and RAASI in these patients. This finding supports the results of previous studies in non-dialysis and dialysis CKD showing a dissociation between sK and potassium intake when estimated on the basis of food questionnaires [40]. It is important to note, however, that factors other than dietary intakes, such as gastrointestinal potassium secretion, or the effect of potassium binders and loop diuretics, that are common treatment options among CKD patients, could induce modifications in the amount of potassium absorbed in the bowel and/or urinary potassium elimination [41,42]. Therefore, measurement of urinary potassium, though being used as surrogate marker of dietary K, is not validated in CKD patients on multiple interventions.

Interestingly, contrasting data cast doubts on the true benefit of restricting potassium in CKD, considering that a diet with a high content of potassium-rich foods, such as plant-based low-protein diets, can be as beneficial for these patients as it is for the general population [38,43]. The role of K intake in the CKD prognosis will be clarified by ongoing trials on the effect potassium supplementation has on CKD progression [44]. An additional original observation regarding nutritional intervention was that UUN and UKV were directly correlated; this finding supports the assumption that it is difficult to attain the benefits of individual nutrients that are linked, such as protein and potassium, when the final goal is to reduce protein intake while maintaining adequate levels of potassium-rich food [45].

The results of this study must be interpreted considering its observational nature; this means that it was possible to assess associations between therapeutic interventions and sK level but not possible to prove a cause-effect relationship; indeed, the treatment options were uncontrolled, and our results may be affected by other issues (CKD stage, heart failure and/or fluid overload). Results moreover may not apply to patients not followed in nephrology clinics and to non-Caucasian patients. Of note, we may have underestimated the prevalence of hyperkalemia, due to availability of only two to three visits in the 12 months of the study. Finally, the cut-off level of sK ≥ 5.0 mEq/L for HK definition might be considered too permissive, thus increasing the prevalence of HK. However, previous studies reported a poor cardiorenal prognosis in CKD patients with sK ≥ 5.0 mEq/L [1,2,3,18]. On the other hand, we performed mixed linear regression analysis to evaluate time-dependent effect on sK of treatment regardless of cut-off used. We also did a sensitivity analysis where sK ≥ 5.5 mEq/L was used as cut-off level, and this analysis showed that, as for the main analysis, the prevalence of sK ≥ 5.5 mEq/L was not modified after 1-year nephrology care, despite the significant increase of therapy.

The strength of this study is that we provide first-time evidence on the relationship between traditional anti-hyperkalemic therapy and sK levels in renal clinics. Although sK levels were not regularly measured as in randomized clinical trials, this real-life study, performed during the everyday clinical practice, does not suffer from the rigid control of the outcomes under investigation that are imposed by interventional protocols and that may limit the generalizability of results.

## 5. Conclusions

In conclusion, this multicentric study suggests that in ND-CKD patients receiving nephrology care, optimal sK control remains unsatisfactory over time in at least one third of CKD patients despite multifactorial anti-HK therapy. Randomized trials have recently demonstrated efficacy and tolerability of new K-binders agents leading to remarkably lower rates of RAASI discontinuation as compared to traditional anti-hyperkalemic therapy [46,47]. These data call for real-world studies conducted in renal clinics, on large number of patients and with long follow up, to verify effectiveness on hard outcomes [48].

## Figures and Tables

**Table 1:** Main clinical features of patients at baseline, overall and by HK subgroup.

	Overall (*n* = 562)	Absent (*n* = 285)	Resolving (*n* = 88)	New Onset (*n* = 93)	Persistent (*n* = 96)	*p*-Value
Age (years)	66.2 ± 14.5	63.6 ± 15.8	68.7 ± 13.8	69.0 ± 12.7	68.9 ± 11.4	0.0004
Male (%)	61.0	63.5	62.5	46.2	66.7	0.014
BMI (Kg/m^2^)	28.7 ± 5.0	28.8 ± 4.8	28.0 ± 4.5	29.0 ± 5.5	28.6 ± 5.5	0.118
Diabetes (%)	33.8	30.5	34.1	34.4	42.7	0.188
CVD (%)	32.9	31.2	34.1	35.5	34.3	0.851
Kidney disease (%)						0.666
HTN	35.2	35.4	37.5	36.6	31.3	
DN	22.2	19.3	22.7	24.7	28.1	
GN	10.5	11.9	10.2	8.6	8.3	
ADPKD	6.1	6.3	2.3	6.5	15.0	
Others	18.3	20.7	19.3	15.1	13.5	
Unknown	7.7	6.3	8.0	8.6	10.4	
CKD stage (%)						<0.001
1–2	13.5	23.5	3.4	5.4	1.0	
3A	19.8	24.6	17.1	18.3	9.4	
3B	29.7	27.0	33.0	35.5	29.2	
4	28.1	20.4	37.5	30.1	40.6	
5	8.9	4.6	9.1	10.8	19.8	
SBP (mmHg)	140 ± 19	139 ± 19	140 ± 22	143 ± 20	141 ± 19	0.247
sAlbumin (g/dL)	4.0 ± 0.5	4.0 ± 0.6	4.0 ± 0.5	4.1 ± 0.5	3.9 ± 0.5	0.192
Hemoglobin (g/dL)	12.8 ± 1.9	13.2 ± 1.8	12.4 ± 2.0	12.6 ± 1.6	11.9 ± 1.7	<0.001
Proteinuria (g/day)	0.47 (0.15–0.86)	0.52 (0.16–0.93)	0.22 (0.10–0.67)	0.26 (0.14–0.75)	0.48 (0.16–0.96)	0.112

Abbreviations: BMI: body mass index; CVD: cardiovascular disease; HTN, hypertensive nephropathy; DN, diabetic nephropathy; GN, glomerulonephritis; ADPKD: autosomal dominant polycystic kidney disease; BP: blood pressure; CKD; chronic kidney disease; SBP, systolic blood pressure.

**Table 2:** Main laboratory parameters at baseline and month-12 visit in the whole cohort and HK subgroups.

	Overall (*n* = 562)	Absent (*n* = 285)	Resolving (*n* = 88)	New Onset (*n* = 93)	Persistent (*n* = 96)	*p*-Value
Serum K (mEq/L) baseline	4.74 ± 0.56	4.38 ± 0.35	5.36 ± 0.37	4.58 ± 0.32	5.37 ± 0.34	--
month 12	4.74 ± 0.54	4.42 ± 0.34	4.56 ± 0.34	5.24 ± 0.22	5.40 ± 0.42	--
*p*-value (baseline vs. final)	0.756	0.131	<0.0001	<0.0001	0.576	
eGFR (ml/min/1.73 m^2^) baseline	39.8 ± 21.8	47.6 ± 24.2	33.5 ± 15.2	33.9 ± 16.2	27.9 ± 13.8	<0.001
month 12	37.9 ± 21.8	45.1 ± 24.3	32.9 ± 16.1	31.6 ± 16.6	27.4 ± 14.1	<0.001
*p*-value (baseline vs. final)	<0.0001	<0.0001	0.612	0.078	0.626	
Serum Bicarbonate (mEq/L) baseline	23.5 ± 5.7	24.7 ± 7.3	23.3 ± 4.2	22.4 ± 2.7	22.1 ± 3.8	0.047
month 12	24.6 ± 3.0	24.8 ± 3.2	24.8 ± 2.3	24.3 ± 3.0	24.4 ± 3.4	0.180
*p*-value (baseline vs. final)	0.014	0.887	0.127	0.002	<0.0001	
Serum Bicarbonate < 22 mEq/L (%) baseline	43.3	30.9	46.2	52.8	60.0	0.006
month 12	22.9	20.2	11.5	30.5	28.9	0.222
*p*-value (baseline vs. final)	<0.0001	0.077	0.007	0.059	0.003	
Salt intake (g/24 h) baseline	8.8 ± 3.6	8.8 ± 3.7	9.1 ± 4.0	8.5 ± 3.1	8.9 ± 3.8	0.636
month 12	8.2 ± 3.4	8.4 ± 3.1	8.3 ± 3.9	7.6 ± 3.9	8.1 ± 3.5	0.358
*p*-value (baseline vs. final)	0.009	0.247	0.295	0.064	0.119	
Urinary K (mEq/24 h) baseline	51.8 ± 23.9	51.9 ± 24.6	47.4 ± 24.1	54.5 ± 23.4	53.3 ± 22.6	0.402
month 12	53.4 ± 27.9	55.8 ± 27.2	47.4 ± 26.3	56.4 ± 33.8	51.0 ± 23.9	0.192
*p*-value (baseline vs. final)	0.370	0.185	0.993	0.630	0.464	
Protein intake (g/24 h/kg) baseline	1.0 ± 0.4	1.0 ± 0.4	0.9 ± 0.3	1.0 ± 0.5	0.9 ± 0.3	0.421
month 12	1.0 ± 0.3	1.0 ± 0.3	0.9 ± 0.3	1.0 ± 0.5	1.0 ± 0.4	0.776
*p*-value (baseline vs. final)	0.591	0.109	0.990	0.853	0.268	

**Table 3:** Mixed effect regression estimating basal determinants of time-dependent serum K.

Variables	Βeta	*p*-Value
CKD stages (%)		
1–2	Ref.	-
3A	0.15 (0.03/0.27)	0.015
3B	0.28 (0.17/0.39)	<0.001
4	0.41 (0.29/0.53)	<0.001
5	0.49 (0.34/0.65)	<0.001
Age (10 years)	0.02 (−0.01/0.04)	0.209
Male	0.05 (−0.02/0.12)	0.165
BMI (kg/m^2^)	0.00 (−0.00/0.01)	0.308
Diabetes (yes/no)	0.08 (0.01/0.16)	0.026
CV disease (yes/no)	0.01 (−0.06/0.08)	0.757
Serum bicarbonate (mEq/L)	−0.01 (−0.02/−0.00)	0.039
Urinary K (mEq/24 h)	0.00 (−0.00/0.00)	0.234
Protein intake (g/day per kg body wt)	0.01 (−0.08/0.10)	0.788
Salt intake (g/day)	−0.00 (−0.01/0.01)	0.576
RAASI (yes/no)	0.05 (−0.03/0.13)	0.212
Non-K sparing diuretics (yes/no)	−0.09 (−0.17/−0.02)	0.014
Bicarbonate supplementation (yes/no)	0.31 (0.15/0.46)	<0.001
K-binders (yes/no)	0.27 (0.03/0.50)	0.025
Time		
Baseline	Ref.	
6 months	0.00 (−0.05/0.05)	0.963
12 months	0.01 (−0.05/0.6)	0.855

Abbreviations: BMI: body mass index; CV: cardiovascular; CKD; chronic kidney disease; RAASI, renin-angiotensin-aldosterone system inhibitors.

## Data Availability

The data underlying this article will be shared on reasonable request to the corresponding author.

## Supplementary Materials

The following are available online at https://www.mdpi.com/2072-6643/13/3/942/s1, Table S1: Basal main clinical features of patients stratified by hyperkalemia status defined as sK ≥ 5.5 mEq/L, Figure S1: Use of Renin-Angiotensin-Aldosterone Inhibitors at baseline (light blue) and at 12-month visit (dark blue) in patients grouped by hyperkalemia status defined as sK ≥ 5.5 mEq/L. * *p* < 0.05 vs. baseline, Table S2: Main therapeutic interventions at baseline and month-12 visit after stratification of patients by sK ≥ 5.5 mmol/L.
