# Supplementary material for: Current Management of Hyperkalemia in Non-Dialysis CKD: Longitudinal Study of Patients Receiving Stable Nephrology Care
Source: Nutrients. 2021 Mar 15;13(3):942. doi: 10.3390/nu13030942 (PMC8000881; doi:10.3390/nu13030942)
Supplement: Supplementary file 1 [file nutrients-13-00942-s001.pdf]

Supplementary

Table S1. Basal main clinical features of patients stratified by hyperkalemia status defined as sK ≥5.5 mEq/L.

	**Absent** (*n* = 468)	**Resolving** (*n* = 48)	**New onset** (*n* = 34)	**Persistent** (n = 12)	p-value
Age (years)	65.6±15.8	69.0±12.3	70.2±14.0	68.0±12.5	0.145
Male (%)	62.2	56.3	58.8	41.7	0.443
BMI (Kg/m2)	28.6±4.8	28.5±6.6	29.2±3.7	31.2±8.3	0.304
Diabetes (%)	31.8	37.5	52.9	41.7	0.072
CVD (%)	31.6	35.4	41.2	50.0	0.373
Kidney disease (%)					0.569
HTN	36.1	27.1	35.3	33.3	
DN	21.8	18.8	32.3	25.0	
GN	10.7	10.4	5.9	16.7	
ADPKD	6.4	6.3	3.0	0.0	
Others	18.4	20.8	17.7	8.3	
Unknown	6.6	16.7	5.9	16.7	
CKD stage (%)					<0.001
1-2	15.8	0	2.9	8.3	
3A	22.2	8.3	5.9	8.3	
3B	30.8	22.9	29.4	16.7	
4	24.2	45.8	50.0	50.0	
5	7.1	22.9	11.8	16.7	
SBP (mmHg)	140±20	139±21	143±18	150±22	0.266
sAlbumin (g/dL)	4.0±0.5	3.8±0.5	4.0±0.5	3.6±0.6	0.007
Hemoglobin (g/dl)	12.9±1.8	12.1±1.8	12.3±1.6	11.3±2.4	<0.001
Proteinuria (g/24h)	0.45 (0.13-0.88)	0.48 (0.16-0.81)	0.46 (0.14-1.50)	0.63 (0.11-2.80)	0.865

Abbreviations: BMI: body mass index; CVD: cardiovascular disease; HTN, hypertensive nephropathy; DN, diabetic nephropathy; GN, glomerulonephritis; ADPKD: autosomal dominant polycystic kidney disease; BP: blood pressure; CKD; chronic kidney disease; SBP, systolic blood pressure.

Figure S1. Use of Renin-Angiotensin-Aldosterone Inhibitors at baseline (light blue) and at 12-month visit (dark blue) in patients grouped by hyperkalemia status defined as sK ≥ 5.5 mEq/L. * p <0.05 vs baseline.

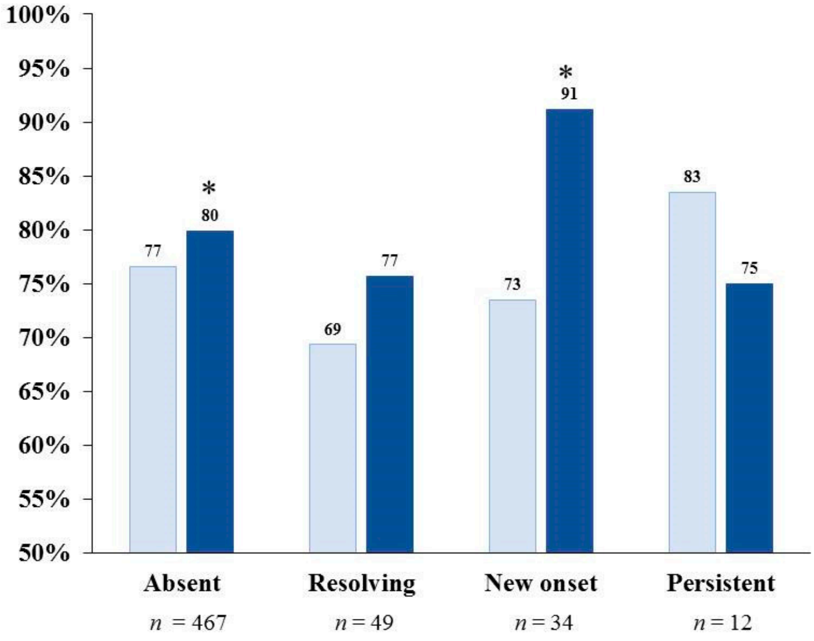

Table S2. Main therapeutic interventions at baseline and month-12 visit after stratification of patients by sK ≥5.5 mmol/L.

		**Absent** (*n* = 467)	**Resolving** (n = 49)	**New onset** (*n* = 34)	**Persistent** (*n* = 12)	p-value
Non-K sparing diuretics (%)	baseline	33.6	28.6	44.1	58.3	0.149
	month 12	41.1	34.7	44.1	75.0	0.085
p-value (baseline vs final)		<0.001	0.180	1.00	0.317	
K-binders (%)	baseline	1.5	4.1	2.9	8.3	0.223
	month 12	5.4	14.3	20.6	33.3	<0.001
p-value (baseline vs final)		<0.001	0.025	0.014	0.083	
Bicarbonate supplements (%)	baseline	2.8	14.3	14.7	25.0	<0.001
	month 12	11.8	12.2	29.4	66.7	<0.001
p-value (baseline vs final)		<0.001	0.706	0.025	0.025	
Salt intake (g/24h)	baseline	8.8±3.7	9.2±3,9	8.6±2.6	7.3±2.4	0.543
	month 12	8.4±3.5	7.9±3.2	6.9±2.0	6.7±1.6	0.059
p-value (baseline vs final)		0.118	0.098	0.0011	0.289	
Protein intake (g/24h/kg)	baseline	1.0±0.4	0.9±0.3	0.8±0.2	1.0±0.4	0.345
	month 12	1.0±0.3	0.9±0.3	0.8±0.3	1.0±0.4	0.238
p-value (baseline vs final)		0.676	0.838	0.823	0.685	
